# Correlation between carbapenem susceptibility in *Pseudomonas aeruginosa* and modified antibiotic heterogeneity index: a multicenter observational study using a surveillance platform

**DOI:** 10.1017/ash.2024.486

**Published:** 2025-01-27

**Authors:** Keisuke Sawada, Takaaki Kato, Shuji Kono, Hiromi Kaneko, Hayato Nakano, Shinobu Inada, Tatsuya Isogawa, Tadahiro Shimizu, Namiko Takahashi, Haruki Takano, Hiroaki Chiba, Makoto Sugimoto, Ryo Inose, Yuichi Muraki, Hideki Araoka

**Affiliations:** 1 Department of Pharmacy, Federation of National Public Service Personnel Mutual Aid Associations Hirakata Kohsai Hospital, Osaka, Japan; 2 Department of Infection Control and Prevention, Federation of National Public Service Personnel Mutual Aid Associations Hirakata Kohsai Hospital, Osaka, Japan; 3 Laboratory of Clinical Pharmacoepidemiology, Kyoto Pharmaceutical University, Kyoto, Japan; 4 National Public Service Personnel Mutual Aid Associations, Tokyo, Japan; 5 Department of Infection Control and Prevention, Federation of National Public Service Personnel Mutual Aid Associations Hiroshima Memorial Hospital, Hiroshima, Japan; 6 Department of Infection Control and Prevention, Federation of National Public Service Personnel Mutual Aid Associations Shinbeppu Hospital, Oita, Japan; 7 Department of Infection Control and Prevention, Federation of National Public Service Personnel Mutual Aid Associations Mishuku Hospital, Tokyo, Japan; 8 Department of Infection Control and Prevention, Federation of National Public Service Personnel Mutual Aid Associations Toranomon Hospital, Tokyo, Japan; 9 Department of Infection Control and Prevention, Federation of National Public Service Personnel Mutual Aid Associations Tohoku Kosai Hospital, Miyagi, Japan; 10 Department of Infection Control and Prevention, Federation of National Public Service Personnel Mutual Aid Associations Yokosuka Kyosai Hospital, Kanagawa, Japan; 11 Department of Infectious Diseases, Federation of National Public Service Personnel Mutual Aid Associations Toranomon Hospital, Tokyo, Japan

## Abstract

**Objective:**

This study focused on exploring the relationship between antimicrobial use indicators, including the modified antibiotic heterogeneity index (mAHI), and the carbapenem susceptibility in *Pseudomonas aeruginosa*.

**Design:**

Survey-based observational study conducted across multiple facilities.

**Setting:**

Public community hospital institutions.

**Methods:**

This survey was conducted in 15 community hospitals in Japan. Indicators, such as the defined daily doses (DDDs), days of therapy (DOTs), antibiotic heterogeneity index (AHI), and mAHI, were analyzed for *P. aeruginosa* carbapenem susceptibility using Spearman’s rank correlation. The predictive accuracies of the AHI and mAHI for carbapenem susceptibility were compared using DeLong’s test for the 2 correlated receiver operating characteristic curves.

**Results:**

No significant correlations were observed between DDDs or DOTs and carbapenem susceptibility. However, a significant correlation was observed between carbapenem susceptibility and the mAHI (*r* = 0.261, *P* = .02), which also demonstrated a higher predictive accuracy for high susceptibility rates than that of the AHI (area under the curve: 0.75 vs 0.58, *p* < .01). The optimal mAHI cutoff value for predicting 90% susceptibility was 0.765, with a sensitivity of 67.7% and specificity of 76.5%.

**Conclusions:**

The mAHI may be a better predictor of carbapenem susceptibility than other commonly used indicators. This study underscores the utility of the mAHI as an effective indicator of antimicrobial usage patterns for managing carbapenem susceptibility in *P. aeruginosa.* Incorporating the mAHI into antimicrobial stewardship programs could enhance the effectiveness of antimicrobial interventions across diverse healthcare settings.

## Background

Antimicrobial resistance (AMR) is a major global public health issue that causes treatment failure, extends therapy duration, and prolongs hospital stays.^1–3^ The spread of AMR in *Pseudomonas aeruginosa*, a common cause of nosocomial infections, is particularly concerning for hospital infection control efforts.^4,5^ In response, Japan aims to lower carbapenem resistance in *P. aeruginosa* to 3% or less by 2027 through its National Action Plan on Antimicrobial Resistance 2023–2027.^6^ To achieve this goal, it is important to optimize the use of antipseudomonal agents.

Antimicrobial stewardship programs (ASPs) are designed to improve the treatment of infections by enhancing prescription patterns and protecting patients from unnecessary antimicrobial use.^7,8^ Hospitals are encouraged to implement ASPs through multidisciplinary teams of physicians and pharmacists.^9,10^ These teams routinely review antimicrobial prescriptions and provide advice to the attending physicians when needed. Monitoring carbapenems and other antipseudomonal agents is a key focus of the ASP team activities.^11,12^

The ASP teams commonly use indicators, such as defined daily doses (DDDs) and days of therapy (DOTs), as factors for monitoring prescriptions.^11,12^ Another indicator, the antibiotic heterogeneity index (AHI), was originally designed to evaluate cyclic antibiotic usage.^13^ This index evaluates the equal distribution of DOTs among 4 types of antibiotics with activity against *P. aeruginosa*: carbapenems, penicillins, cephalosporins, and quinolones. The modified (updated) AHI (mAHI) was proposed in 2023, which limits quinolone DOTs to 10% of the total while maintaining 30% for each of the other 3 types.^14^ The relationship between these indicators and drug susceptibility in *P. aeruginos*a has been confirmed in several single-facility studies.^14–17^

The use of antipseudomonal agents varies depending on the size and geographical location of the hospital.^5,18^ Although multicenter studies employing DDDs or DOTs have shown a relationship with *P. aeruginosa* susceptibility rates, these studies were often limited to specific regions.^17,19^ Moreover, there are few multicenter studies on the AHI and mAHI, and it is unclear how differences in facilities and regional characteristics affect these results. Therefore, the applicability of these indicators in various settings is yet to be fully proven. This study aimed to explore the relationship between indicators of antimicrobial use and carbapenem susceptibility in *P. aeruginosa* through a multicenter survey, considering the differences between institutions and regions in community hospitals across Japan.

## Methods

### Study setting

In this survey, we examined the correlation between carbapenem susceptibility in *P. aeruginosa* and several indicators of antimicrobial use (DDDs, DOTs, AHI, and mAHI). This study was conducted in 15 community hospitals associated with the Federation of National Public Service Personnel Mutual Aid Associations in Japan. These facilities receive additional healthcare reimbursements for type 1 infection prevention and control,^20^ which represents Japan’s highest standards for infection control. This designation ensures that each facility maintains an infection control team, including certified nurses, conducts regular in-hospital training programs, and implements antimicrobial monitoring systems through ASP teams.

### Data source

Data were obtained from the Japanese Surveillance for Infection Prevention and Healthcare Epidemiology (J-SIPHE), managed by the AMR Clinical Reference Center since 2019.^21^ J-SIPHE serves as a web platform that collects detailed information on infection control and AMR from its member facilities. Regularly collected data include the quantity and duration of antimicrobial usage, identification and drug susceptibility of the isolated bacteria, and the hours and activities involved in ASPs at each hospital. The participating facilities receive feedback, including anonymized multicenter comparisons, as part of the information-sharing process through approved groups on the platform. The dataset used in this study was obtained from 15 hospitals using a specific feature of the J-SIPHE platform for group-based data extraction.

### Definition

#### Implementation of ASPs

Data were collected from 15 facilities in March 2023. This information included the facility’s location, the number of beds available, whether it had an on-site bacteriology lab, and actions taken toward implementing ASPs. The number of team members involved in ASPs, their qualifications, and hours dedicated to each occupation category were counted. To standardize these work hours, they were converted into full-time equivalents (FTEs), considering a 40-hour workweek to be 1.0. The assessment of ASP efforts at each facility was categorized into 3 groups based on the following definitions:^10,22–24^

1. Prior Authorization System: Requires approval before certain antimicrobials can be prescribed.
2. Notification System: Requires a report after prescribing certain antimicrobials, aiding in monitoring and auditing.
3. Prospective Audit and Feedback (PAF): The ASP team monitors the prescription of a target drug and intervenes early to ensure its appropriate use.

#### Antimicrobial use indicators

Data on the utilization and duration of injectable antimicrobial prescriptions between April 2020 and March 2023 were collected. Antimicrobial use indicators were calculated every 6 months, using the categories and formulae shown in Table 1.

**Table 1.** Definitions of antimicrobial use indicators and categories

	Definition
Indicators	
DDDs	(Total amount per antimicrobial/DDD per antimicrobial) × (100/bed-days)
DOTs	(Total number of days per antimicrobial) × (100/bed-days)
AHI	1 – {2/3×Σ|0.25 – (DOTs of each class/total DOTs of all classes) |}
mAHI	1 – {2/3×Σ|Coefficient ^a^ – (DOTs of each class/total DOTs of all classes) |}
Categories	
Carbapenems	Meropenem, doripenem, biapenem, imipenem/cilastatin, imipenem/cilastatin/relebactam, panipenem/betamipron
Penicillins	Piperacillin, piperacillin/tazobactam
Cephalosporins	Ceftazidime, cefoperazone/sulbactam, cefepime, cefozopran, cefpirome, ceftorosan/tazobactam
Quinolones	Ciprofloxacin, pazufloxacin, levofloxacin

Note. AHI, antibiotic heterogeneity index; DDDs, defined daily doses; DOTs, days of therapy; mAHI, modified antibiotic heterogeneity index.

a Coefficient: 0.1 for quinolones, 0.3 for carbapenems, penicillins, and cephalosporins.

#### Carbapenem susceptibility in *P. aeruginosa*

Bacterial culture test results from April 2020 to March 2023 were analyzed. The number of *P. aeruginosa* isolates identified and meropenem susceptibility were recorded every 6 months. Cultures were obtained from various samples, including blood, sputum, urine, spinal fluid, amniotic fluid, dural fluid, pus from abscesses and wounds, bile, endotracheal aspirates, drainage fluids, joint fluids, and stool. If *P. aeruginosa* was found more than once in the same patient, the strains found within 3 months of the first visit were considered the same strain. A strain was determined as susceptible or resistant according to the Clinical and Laboratory Standards Institute M100-S22 (2012) criteria embedded in the J-SIPHE surveillance platform, with intermediate resistance classified as resistant. The breakpoints for meropenem against *P. aeruginosa* remained unchanged through M100-S33 (2023), ensuring consistent interpretation of susceptibility throughout the study period.^25^ Following the J-SIPHE platform specifications, data were treated as missing when the number of *P. aeruginosa* isolates was <10 in a specified period.

### Statistical analysis

Each antimicrobial use indicator and the carbapenem susceptibility rate for every 6-month period were plotted for each hospital. Spearman’s rank correlation coefficient was used to analyze the relationships between them. For sensitivity analysis, we performed additional analyses after removing outliers identified using the Smirnov–Grubbs test and conducted beta regression to account for the upper limit (100%) of susceptibility rates. To determine cutoff values for AHI and mAHI predicting 80% or 90% carbapenem susceptibility, the area under the curve (AUC) analysis with receiver operating characteristic (ROC) curves was used. The accuracy of predicting carbapenem susceptibility using AHI and mAHI was compared using DeLong’s test for the 2 correlated ROC curves. The optimal cutoff values were determined using Youden’s index (sensitivity + specificity − 1).

The hospitals were divided into 3 groups based on mAHI tertiles. The differences in hospital functions, composition of the ASP team, and activities among these groups were explored using the Kruskal–Wallis test for multiple comparisons. For between-group comparisons, the Mann–Whitney test was applied with Bonferroni correction for multiple testing. Fisher’s exact test was used for categorical variables.

All statistical analyses were performed using R version 4.4.2 with Rcmdr (version 2.9-5) and betareg (version 3.2-1) packages for sensitivity analyses. A two-sided *P* value < .05 was considered statistically significant.

### Ethical considerations

This study was approved by the Ethics Committee of Hirakata Kosai Hospital (2023-016), and all participating institutions provided informed consent. Our study relied on an antimicrobial and bacterial database separate from patient details; therefore, direct consent from individual patients was deemed unnecessary.

## Results

### Implementation of ASPs

**Figure 1.**
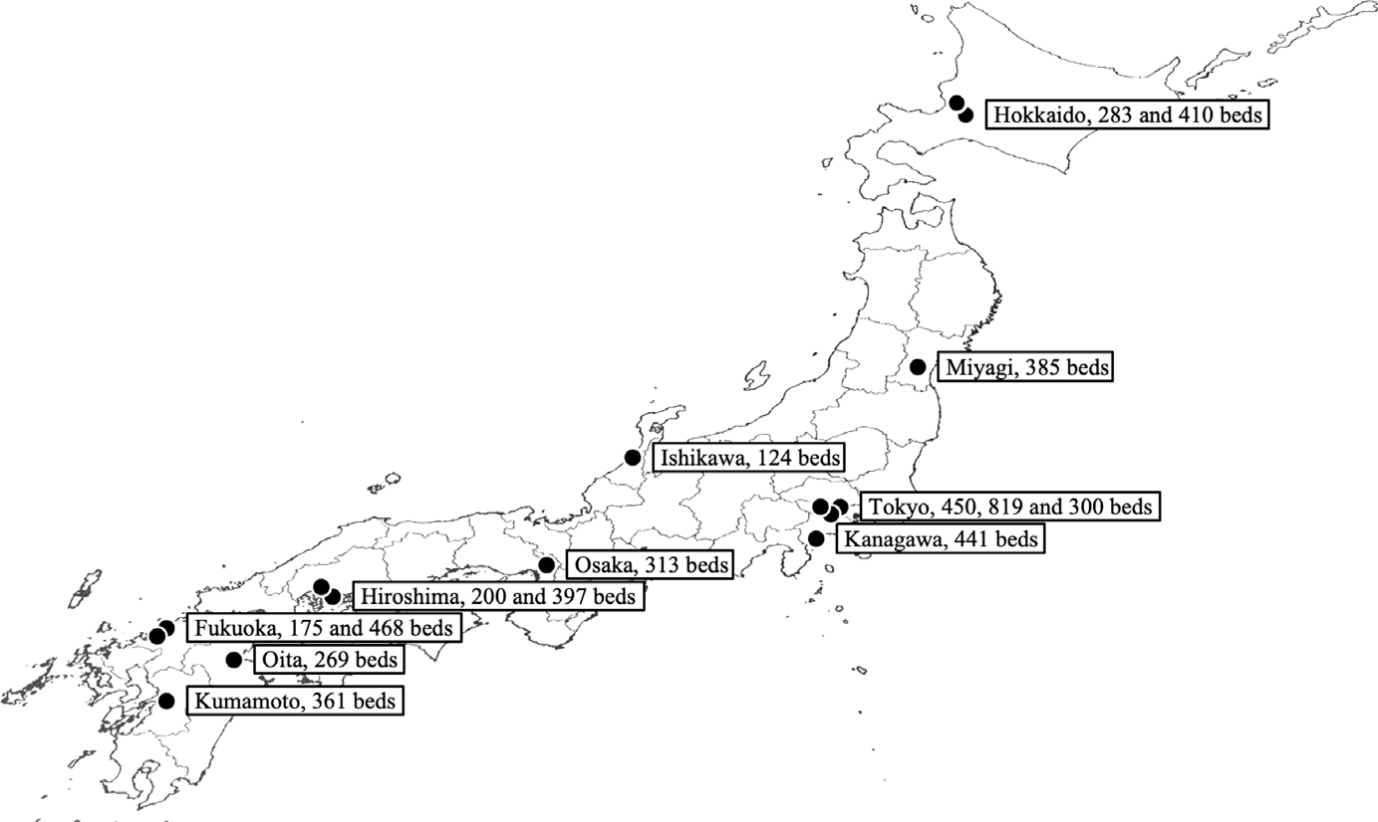
Location and bed capacity of surveyed facilities. *Note*: Created by editing the Digital Topographic Map 25000 of Geospatial Information Authority of Japan.

Among the 15 facilities surveyed, the median number of beds was 361 (interquartile range: 273–425), and 10 facilities (66.7%) had an in-hospital bacteriology laboratory (Figure 1). Physicians were the most common members of the ASP teams (median = 2), followed by nurses, laboratory technicians, and pharmacists. Pharmacists had the highest number of FTEs (0.50) compared with that of other occupations (0.10–0.15) (Table 2). All facilities had a notification system for carbapenems, and PAF was also practiced in 66.7% of the facilities (Table 3).

**Table 2.** Composition of members and full-time equivalents (FTEs) in antimicrobial stewardship programs teams

	Physicians	Nurses	Laboratory technicians	Pharmacists
Participants	2.0 (1.0–2.0)	1.0 (1.0–2.0)	1.0 (1.0–2.0)	1.0 (1.0–2.5)
Qualified	1.0 (0.5–1.0)	1.0 (1.0–1.5)	0 (0–1.0)	1.0 (1.0–1.0)
FTEs	0.10 (0.03–0.20)	0.15 (0.04–0.40)	0.10 (0.03–0.36)	0.50 (0.23–0.99)

Note. FTEs, full-time equivalents, 40 hours per week considered as 1.0.

Values are expressed as medians (interquartile ranges).

### Correlation between *P. aeruginosa* carbapenem susceptibility rates and antimicrobial use indicators

**Figure 2.**
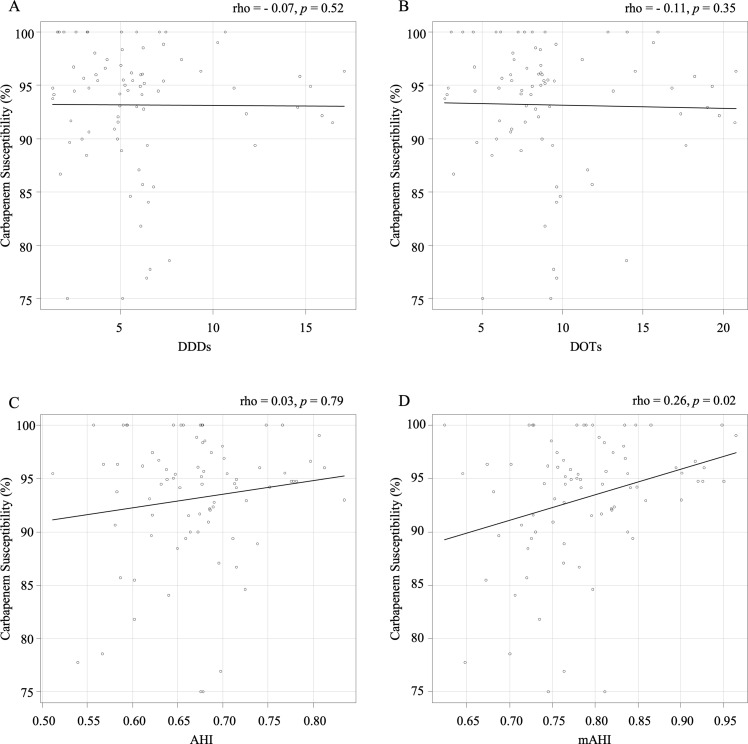
Correlation between various antimicrobial usage indicators and the carbapenem susceptibility rate in *P. aeruginosa*. A: Carbapenem susceptibility rate versus DDDs. B: versus DOTs. C: versus AHI. D: versus mAHI. *Note:* AHI, antibiotic heterogeneity index; DDDs, defined daily doses; DOTs, days of therapy; mAHI, modified antibiotic heterogeneity index.

Data were collected from 15 facilities for 6 periods each, totaling 79 periods after excluding 11 periods with missing drug susceptibility data. The analysis showed that the DDDs and DOTs of all antipseudomonal agents were not significantly correlated with the carbapenem susceptibility rates in *P. aeruginosa* for either indicator (Figure 2A, 2B). This was also true when specific types of antimicrobials were analyzed (Supplementary Figure 1). Although no significant correlation was found with the AHI, a significant correlation (*ρ* = 0.261, *P* = .02) was observed between the mAHI and carbapenem susceptibility (Figure 2C, 2D). These relationships remained consistent in sensitivity analyses using both outlier removal and beta regression (Supplementary Tables S1 and S2).

### Comparison of the accuracy of AHI and mAHI in predicting carbapenem susceptibility

**Figure 3.**
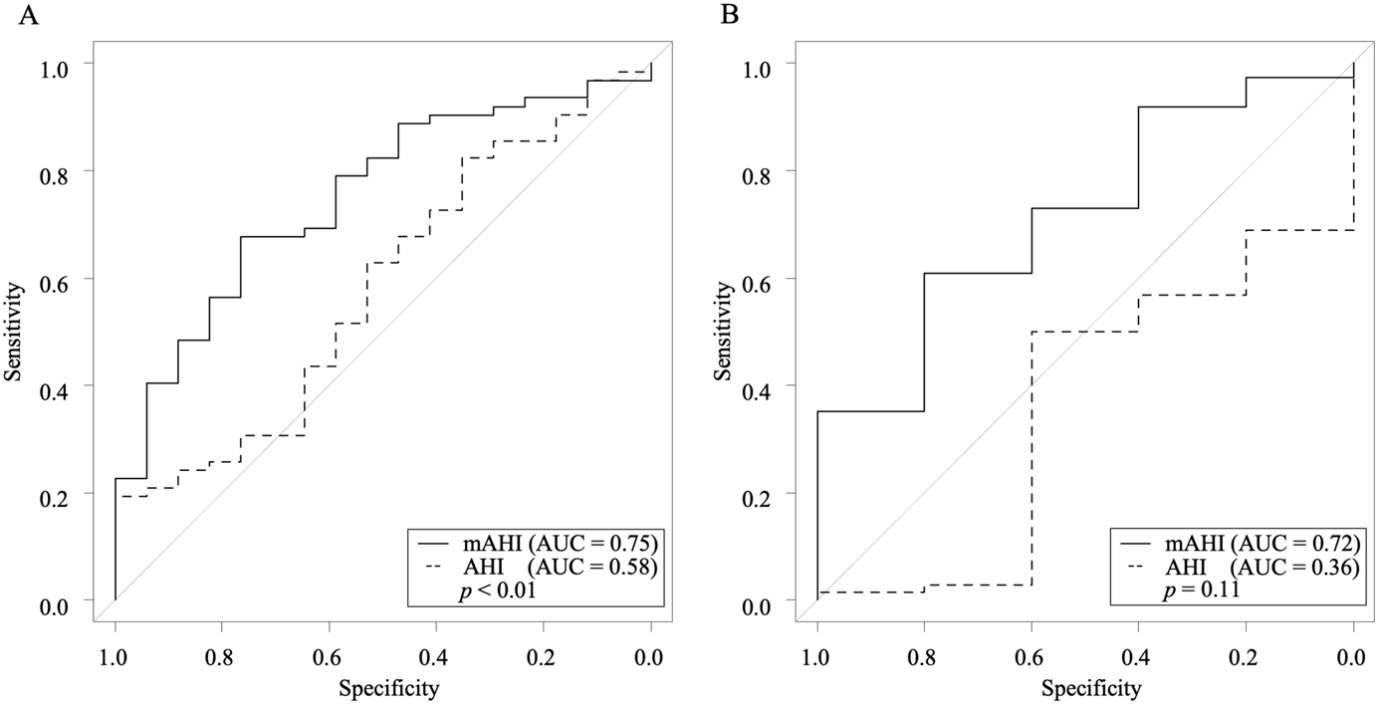
Comparison of the predictive accuracy of mAHI and AHI for carbapenem susceptibility rates in *P. aeruginosa*. A: 90% susceptibility. B: 80% susceptibility. *Note:* AHI, antibiotic heterogeneity index; AUC, area under the curve, mAHI, modified antibiotic heterogeneity index.

The mAHI demonstrated significantly higher accuracy than AHI in predicting a carbapenem susceptibility rate of 90% in *P. aeruginosa* (AUC: 0.75 vs 0.58, *P* < .01) (Figure 3A). However, there was no significant difference in predictive accuracy for an 80% susceptibility rate between the mAHI and AHI (AUC: 0.72 vs 0.36, *P* = .11) (Figure 3B). The mAHI cutoff values predicting 90% and 80% carbapenem susceptibility were both 0.765, with sensitivities of 67.7% and 60.8% and specificities of 76.5% and 80.0%, respectively.

### Differences in ASP implementation based on mAHI cutoff values

Facilities were divided into 3 groups based on mAHI values: low (<0.765), middle (0.765–0.825), and high (>0.825). No statistically significant differences in ASP implementation were observed among these groups (Table 4). Although not statistically significant, the high mAHI group showed higher PAF implementation rates for carbapenems (80.0%), penicillins (80.0%), and quinolones (60.0%). In this group, FTEs were lower for all occupations (0.03) except pharmacists (0.8).

**Table 3.** Surveillance systems for prescribing antipseudomonal agents

	Carbapenems	Penicillins	Cephalosporins	Quinolones
Prior authorization	0 (0 %)	0 (0 %)	1 (6.7 %)	1 (6.7 %)
Notification	15 (100 %)	8 (53.3 %)	6 (40.0 %)	3 (20.0 %)
PAF	10 (66.7 %)	9 (60 %)	7 (46.7 %)	5 (33.3 %)

Note. PAF, prospective audit and feedback.

**Table 4.** Differences in ASPs implementation based on the modified antibiotic heterogeneity index (mAHI) values

	Low-mAHI (<0.765) (n = 5)	Middle-mAHI (0.765–0.825) (n = 5)	High-mAHI (>0.825) (n = 5)	*P* value
Bed size^a^	361 (283–410)	368 (263–450)	313 (300–396)	0.878
In-hospital bacteriology laboratory	3 (60.0 %)	4 (80.0 %)	3 (60.0 %)	1.00
Participants^a^				
Physicians	2 (1–2)	2 (2–2)	1 (1–1)	0.172
Nurses	1 (1–2)	2 (1–2)	1 (1–1)	0.459
Laboratory technicians	2 (1–2)	1 (1–2)	1 (1–1)	0.090
Pharmacists	2 (2–3)	1 (1–1)	1 (1–3)	0.193
Qualified^a^				
Physicians	1 (1–1)	1 (1–1)	1 (0–1)	0.533
Nurses	1 (1–1)	1 (1–2)	1 (1–1)	0.728
Laboratory technicians	0 (0–0)	1.0 (0–1)	0 (0–0)	0.326
Pharmacists	1 (1–1)	1 (1–1)	1 (1–1)	0.842
FTEs^a^	2.48 (1.56–2.90)	1.13 (0.65–1.26)	1.03 (0.28–1.03)	0.084
Physicians	0.20 (0.10–0.20)	0.10 (0.05–0.23)	0.03 (0.03–0.05)	0.116
Nurses	0.20 (0.13–1.00)	0.30 (0.15–0.50)	0.03 (0.03–0.05)	0.062
Laboratory technicians	0.33 (010–0.50)	0.10 (0.05–0.40)	0.03 (0.03–0.03)	0.069
Pharmacists	0.98 (0.50–1.13)	0.33 (0.15–0.40)	0.80 (0.19–1.00)	0.195
Prior authorization or notification				
Carbapenems	5 (100 %)	5 (100 %)	5 (100 %)	NA
Penicillins	3 (60.0 %)	2 (40.0 %)	3 (60.0 %)	1.00
Cephalosporins	2 (40.0 %)	3 (60.0 %)	2 (40.0 %)	1.00
Quinolones	1 (20.0 %)	1 (20.0 %)	2 (40.0 %)	1.00
PAF				
Carbapenems	2 (40.0 %)	4 (80.0 %)	4 (80.0 %)	0.50
Penicillins	2 (40.0 %)	3 (60.0 %)	4 (80.0 %)	0.80
Cephalosporins	3 (60.0 %)	1 (20.0 %)	2 (40.0 %)	0.80
Quinolones	1 (20.0 %)	1 (20.0 %)	3 (60.0 %)	0.50

Note. ASP, antimicrobial stewardship program; FTEs, full-time equivalents, 40 hours per week considered as 1.0; PAF, prospective audit and feedback.

a Median values (interquartile ranges).

## Discussion

This study, conducted in 15 community hospitals in Japan, revealed a significant correlation between the mAHI and carbapenem susceptibility in *P. aeruginosa.* The cutoff value of the mAHI for predicting susceptibility rates of 80% and 90% was 0.765. In the high mAHI group (>0.825), despite high PAF implementation rates, FTEs remained low for all occupations except pharmacists.

In this survey conducted across multiple facilities in various regions, no statistical correlation was found between the DDDs or DOTs of antipseudomonal agents and carbapenem susceptibility in *P. aeruginosa*. This finding contrasts with the results of other studies conducted in single facilities or specific regions.^15,17,19,26−30^ Antipseudomonal agents are widely used in departments specializing in the care of immunocompromised patients, such as those in intensive care units or undergoing transplants or tumor treatments.^31,32^ A significant difference in the amount and duration of use of these antimicrobials between tertiary and community hospitals has been reported.^33,34^ Moreover, the drug susceptibility of *P. aeruginosa* varies significantly across geographical regions.^14^ Although DDDs and DOTs are suitable indicators for assessing temporal trends within a single facility, they may not be appropriate for comparing facilities of different sizes and locations or for setting common target values.

Other single-facility studies found a correlation between carbapenem susceptibility in *P. aeruginosa* and both the AHI and mAHI; however, it was not clear which was superior.^13,16^ In this multi-facility study, the mAHI showed a larger AUC value than the AHI for predicting carbapenem susceptibility. This difference may reflect the underlying assumptions of each index: The AHI assumes the uniform use of carbapenems, penicillins, cephalosporins, and quinolones,^14,16^ while the mAHI assumes a 10% limit for the use of quinolones, with the remaining 3 classes used uniformly at 30%.^32,33^ The use of quinolones as injectable antibiotics is reported to be 23% in the United States and 12.4% in Japan.^16,33,34^ Therefore, in Japan, using the mAHI as a benchmark may reflect actual usage rates more closely than using the AHI. Despite potential confounding factors, this study found a statistically significant correlation between the carbapenem susceptibility rate of *P. aeruginosa* and mAHI, suggesting that mAHI may be a more suitable indicator for facilities in Japan. Further research is needed to investigate how the relationship between mAHI and susceptibility is maintained across different ranges of antimicrobial use (ie, high and low DOTs).

Regarding the differences in ASP implementation among the 3 mAHI groups, although the number of facilities was insufficient for statistical evaluation, the high mAHI group showed the highest rates of PAF implementation for antipseudomonal agents excluding cephalosporins. The PAF reduces the selection rate and usage days of the targeted drugs.^22,23^ Overreliance on a single antimicrobial agent can lead to drug resistance.^32,35^ Therefore, by implementing PAF for high-DOT antipseudomonal agents, it may be possible to increase the mAHI and contribute to the suppression of resistance development. Notably, the high mAHI group achieved better balanced antimicrobial use despite lower FTE values, except for pharmacists. This suggests that effective ASP implementation may depend more on well-structured PAF interventions led by pharmacists than on the total number of FTEs. Focusing on targeted PAF activities might be a key strategy for achieving balanced antimicrobial use and maintaining high mAHI values.

A study conducted using real-world data requires careful interpretation of findings. This study shows that DDDs and DOTs do not correlate with carbapenem susceptibility in *P. aeruginosa*. However, this is limited to actual usage. Therefore, the assumption that DDDs and DOTs can increase indefinitely without adverse effects as long as the mAHI is maintained is incorrect. From the perspective of individual facilities, efforts to reduce the DOTs of antipseudomonal agents should be considered. Moreover, it is important to compare the mAHI across multiple facilities to ensure the balanced use of these antimicrobials without bias toward specific drugs.

This study has 3 major limitations related to its data source. First, the J-SIPHE database was compiled based on the information of medical fee reimbursement, which may contain slight discrepancies from the actual dosages. For instance, injectable antibiotics prescribed by dentists, which are not included in this data source, may have resulted in an underestimation of dosages. Second, although all 15 facilities in this study met the standards for type 1 infection prevention and control reimbursement, ensuring a consistent level of infection control practices, quantitative indicators, such as hand hygiene compliance rates, were not included in our analysis. Third, we could not distinguish between community- and hospital-acquired *P. aeruginosa* infections. Although hospital-acquired infections are typically defined as those occurring 48 hours or more after admission, the J-SIPHE platform does not include information about the timing of bacterial cultures. The emergence of antimicrobial-resistant organisms is influenced by 3 key factors: antimicrobial use patterns, infection control practices, and community resistance rates. Future large-scale studies using Japanese surveillance systems would benefit from mechanisms to collect and differentiate these critical pieces of information.

This study revealed that the mAHI, which serves as an indicator of antimicrobial use, correlates with carbapenem susceptibility in *P. aeruginosa* across various hospital settings. Evaluating the mAHI as part of the ASPs is expected to be beneficial for managing the proper use of antimicrobials.

## Supporting information

Sawada et al. supplementary material Sawada et al. supplementary material

## Data Availability

We obtained our data from J-SIPHE, operated by the AMR Clinical Reference Center. Therefore, these data are not publicly available. If other researchers wish to use the data, they need to apply to the AMR Clinical Reference Center.

## Supplementary material

For supplementary material accompanying this paper visit http://doi.org/10.1017/ash.2024.486. click here to view supplementary material
