# Supplementary material for: Correlation between carbapenem susceptibility in Pseudomonas aeruginosa and modified antibiotic heterogeneity index: a multicenter observational study using a surveillance platform
Source: Antimicrob Steward Healthc Epidemiol. 2025 Jan 27;5(1):e21. doi: 10.1017/ash.2024.486 (PMC11795433; doi:10.1017/ash.2024.486)
Supplement: Sawada et al. supplementary material [file S2732494X24004868sup001.docx]

**Supplemental Material**

**
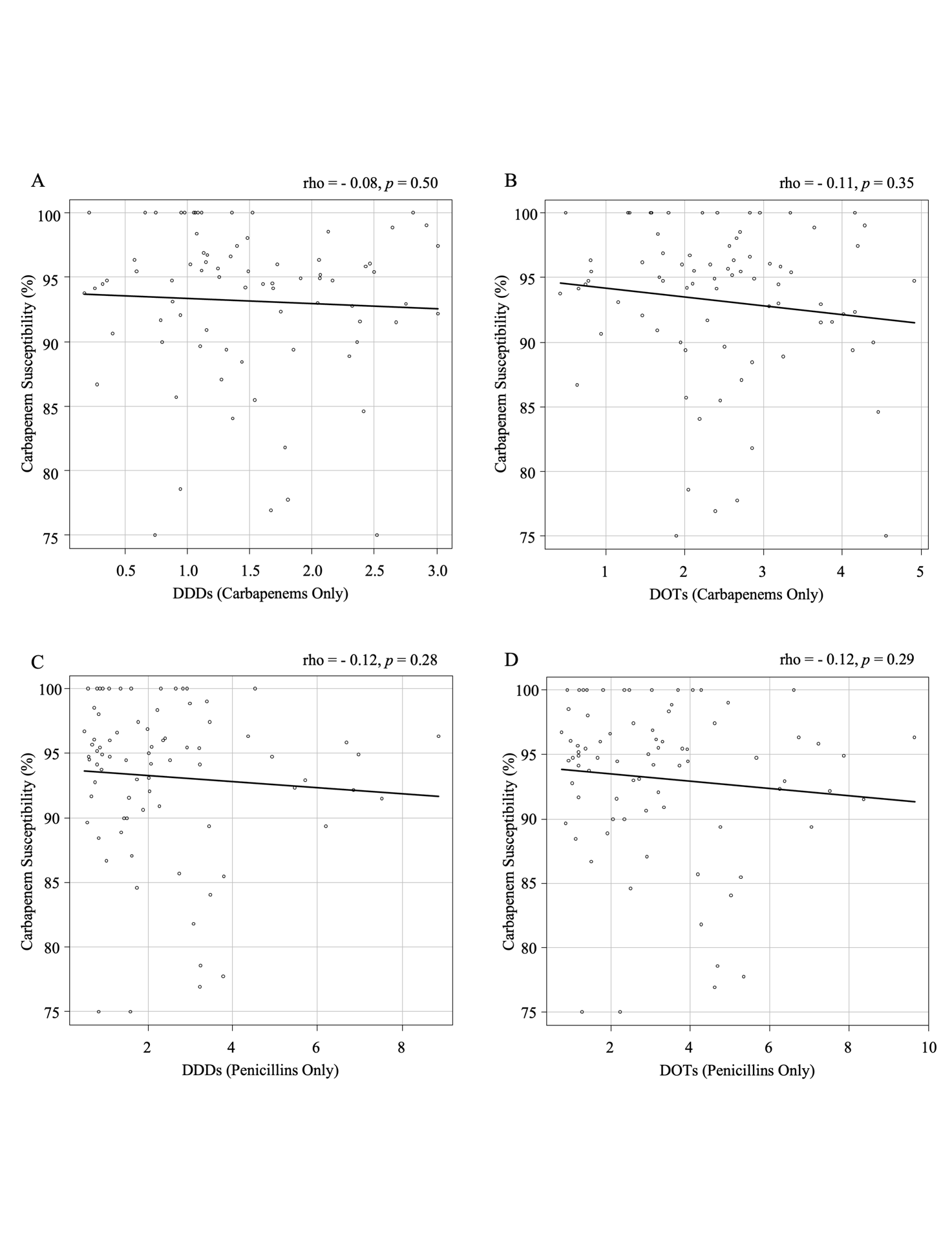
**

**
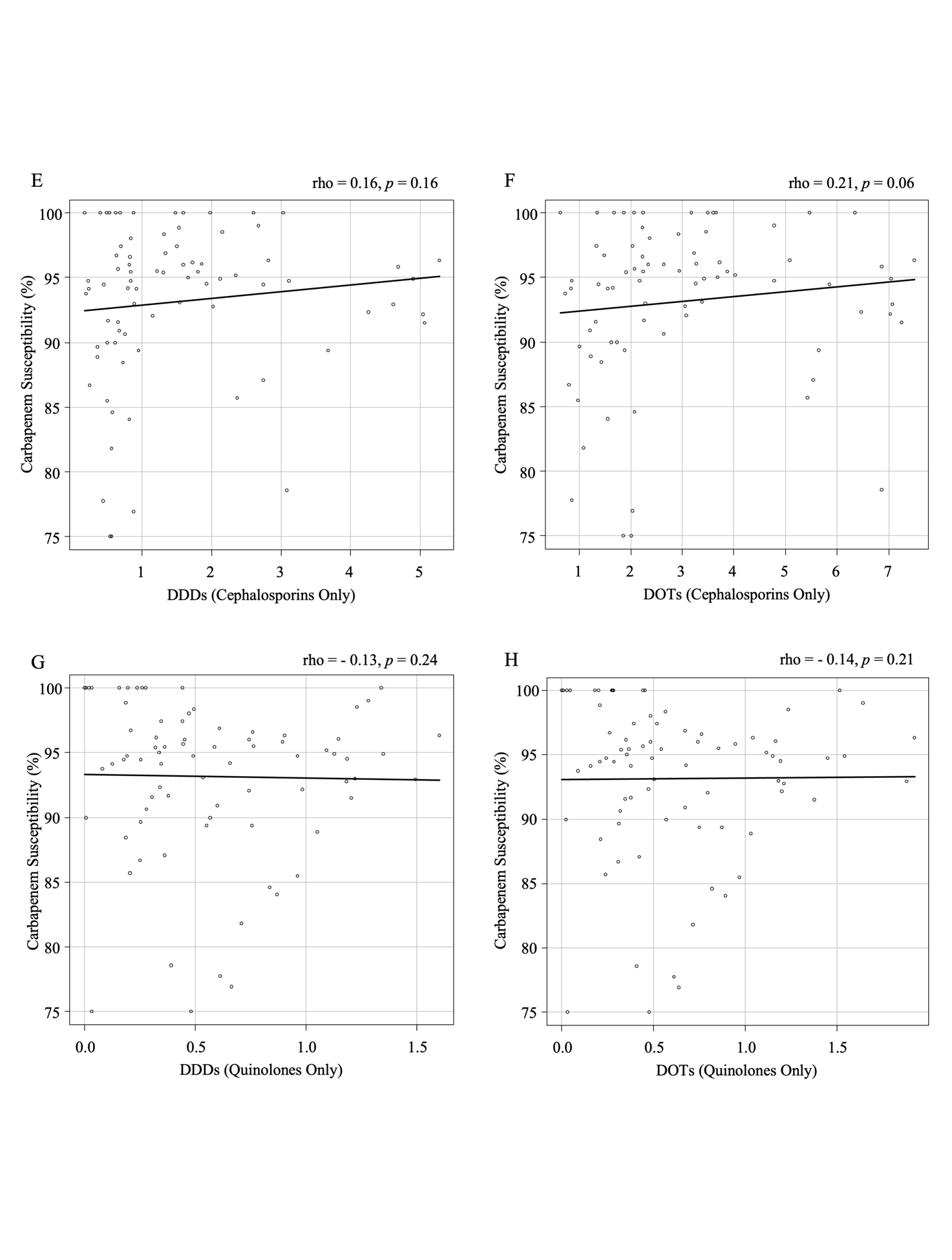
**

Supplementary Figure 1. Correlation between Various Antimicrobial Usage Indicators and the Carbapenem Susceptibility Rate in *Pseudomonas aeruginosa*

A: Carbapenem Susceptibility Rate vs DDDs of Carbapenems. B: vs DOTs of Carbapenems. C: vs DDDs of Penicillins. D: vs DOTs of Penicillins. E: vs DDDs of Cephalosporins. F: vs DOTs of Cephalosporins. G: vs DDDs of Quinolones. H: vs DOTs of Quinolones.

DDDs: defined daily doses; DOTs: days of therapy.

Supplementary Table 1. Sensitivity Analysis of Correlation between Antimicrobial Use Indicators and Carbapenem Susceptibility Rate After Outlier Removal

| Indicators | Outliers | Spearman's rho | p-value |
| --- | --- | --- | --- |
| DDDs | None | -0.073 | 0.524 |
| Carbapenems | None | -0.078 | 0.497 |
| Penicillins | Yes (8.851) | -0.142 | 0.214 |
| Cephalosporins | None | 0.160 | 0.160 |
| Quinolones | None | -0.134 | 0.238 |
| DOTs | None | -0.107 | 0.348 |
| Carbapenems | None | -0.107 | 0.350 |
| Penicillins | None | -0.122 | 0.286 |
| Cephalosporins | None | 0.210 | 0.064 |
| Quinolones | None | -0.142 | 0.213 |
| AHI | None | 0.031 | 0.789 |
| mAHI | None | 0.261 | 0.020 |

DDDs: defined daily doses; DOTs: days of therapy; AHI: antibiotic heterogeneity index; mAHI: modified antibiotic heterogeneity index.

Outliers were detected using Smirnov–Grubbs test. Only mAHI showed significant positive correlation.

Supplementary Table 2. Sensitivity Analysis: Beta Regression between Antimicrobial Use Indicators and Carbapenem Susceptibility Rate

| Indicators | Estimate (Std. Error) | Phi coefficient | p-value |
| --- | --- | --- | --- |
| DDDs | -0.006 (0.020) | 28.013 | 0.782 |
| Carbapenems | -0.046 (0.105) | 28.088 | 0.660 |
| Penicillins | -0.029 (0.039) | 29.233 | 0.459 |
| Cephalosporins | 0.042 (0.060) | 28.075 | 0.483 |
| Quinolones | -0.104 (0.196) | 27.511 | 0.598 |
| DOTs | -0.006 (0.017) | 28.124 | 0.744 |
| Carbapenems | -0.073 (0.072) | 28.793 | 0.309 |
| Penicillins | -0.032 (0.035) | 29.059 | 0.372 |
| Cephalosporins | 0.038 (0.043) | 28.360 | 0.376 |
| Quinolones | -0.053 (0.172) | 27.780 | 0.760 |
| AHI | 1.138 (1.173) | 29.726 | 0.332 |
| mAHI | 2.644 (1.019) | 34.080 | 0.009 |

DDDs: defined daily doses; DOTs: days of therapy; AHI: antibiotic heterogeneity index; mAHI: modified antibiotic heterogeneity index.

Estimate represents the regression coefficient with logit link. Std. Error is shown in parentheses. Phi coefficient indicates the precision parameter of the beta regression model, with values above 30 indicating high precision (low variance). Only mAHI showed significant positive association.
